# The Gridlock transcriptional repressor impedes vertebrate heart regeneration by restricting expression of lysine methyltransferase

**DOI:** 10.1242/dev.190678

**Published:** 2020-09-28

**Authors:** Peilu She, Huifang Zhang, Xiangwen Peng, Jianjian Sun, Bangjun Gao, Yating Zhou, Xuejiao Zhu, Xueli Hu, Kaa Seng Lai, Jiemin Wong, Bin Zhou, Linhui Wang, Tao P. Zhong

**Affiliations:** 1 State Key Laboratory of Genetic Engineering, School of Life Sciences, Zhongshan Hospital, Fudan University, Shanghai, 200438, China; 2 Shanghai Key Laboratory of Regulatory Biology, Institute of Molecular Medicine, School of Life Sciences, East China Normal University, Shanghai, 200241, China; 3 Institute of Biochemistry and Cell Biology, Chinese Academy of Sciences, Shanghai, 200031, China; 4 Department of Urology, Shanghai Changzheng Hospital, Shanghai, 200003, China

**Keywords:** Grl, Hey2, Heart regeneration, Cardiomyocyte proliferation, Lysine methyltransferase, Zebrafish

## Abstract

Teleost zebrafish and neonatal mammalian hearts exhibit the remarkable capacity to regenerate through dedifferentiation and proliferation of pre-existing cardiomyocytes (CMs). Although many mitogenic signals that stimulate zebrafish heart regeneration have been identified, transcriptional programs that restrain injury-induced CM renewal are incompletely understood. Here, we report that mutations in *gridlock* (*grl*; also known as *hey2*), encoding a Hairy-related basic helix-loop-helix transcriptional repressor, enhance CM proliferation and reduce fibrosis following damage. In contrast, myocardial *grl* induction blunts CM dedifferentiation and regenerative responses to heart injury. RNA sequencing analyses uncover Smyd2 lysine methyltransferase (KMT) as a key transcriptional target repressed by Grl. Reduction in Grl protein levels triggered by injury induces *smyd2* expression at the wound myocardium, enhancing CM proliferation. We show that Smyd2 functions as a methyltransferase and modulates the Stat3 methylation and phosphorylation activity. Inhibition of the KMT activity of Smyd2 reduces phosphorylated Stat3 at cardiac wounds, suppressing the elevated CM proliferation in injured *grl* mutant hearts. Our findings establish an injury-specific transcriptional repression program in governing CM renewal during heart regeneration, providing a potential strategy whereby silencing Grl repression at local regions might empower regeneration capacity to the injured mammalian heart.

## Abstract (precis)

**Highlighted Article:** Novel mechanisms of the Grl-Smyd2 network govern vertebrate CM renewal and heart regeneration, which might be relevant in developing strategies for regeneration interventions in humans.

## INTRODUCTION

The human heart has limited capacity to regenerate new cardiac muscle after myocardial injury. Instead, impaired myocardium is replaced by fibrotic scar, eventually leading to heart failure (Tzahor and Poss, 2017; Xin et al., 2013). By contrast, zebrafish heart possesses natural regeneration capacity by dedifferentiation and proliferation of pre-existing cardiomyocytes (CMs) after injury (Jopling et al., 2010; Kikuchi et al., 2010; Li et al., 2015). Hearts of mice can regenerate if injured in the few days after birth, which coincides with the transient capacity for CM proliferation (Porrello et al., 2011). The limited regenerative potential in the adult mammalian heart can be ascribed to cell-intrinsic barriers or molecular blocks that prevent CMs from entering proliferation and completing cytokinesis after damage (González-Rosa et al., 2018; Li et al., 2015; Tzahor and Poss, 2017). Various mitogenic factors and signaling pathways have been identified as initiating and achieving heart regeneration (Fang et al., 2013; Han et al., 2019; Harrison et al., 2019; Kikuchi et al., 2011; Marin-Juez et al., 2016; Mohamed et al., 2018; Monroe et al., 2019; Wu et al., 2016; Zhao et al., 2019). However, transcriptional programs and epigenetic signaling that serve as roadblocks to limit injury-induced CM renewal are incompletely understood.

Gridlock (Grl; also known as Hey2) is a basic helix-loop-helix (bHLH) transcriptional repressor belonging to the Hesr (Hairy/Enhancer-of-split related) family (also known as Hey or Hrt family) containing Hey1, Grl and HeyL, which play important functions via diverse mechanisms involving vascular, myocardial, endocardial and neuronal tissues during development (Fischer et al., 2002; Nakagawa et al., 1999; Sakamoto et al., 2003; Zhong et al., 2000). Hey proteins mediate transcriptional repression by directly binding an E-box DNA motif, and can repress GATA-mediated transcription via physically interacting with GATA factors (Heisig et al., 2012; Jia et al., 2007; Kathiriya et al., 2004). We and others previously reported that Grl and Hey1 regulate arterial-venous differentiation in zebrafish and mice (Fischer et al., 2004; Zhong et al., 2001). Grl also contributes to zebrafish heart development by limiting expansion of cardiac progenitor cells (CPC) and embryonic CMs (Gibb et al., 2018; Jia et al., 2007). During cardiovascular development, Hey family genes function in response to Notch signaling, including atrioventricular boundary formation, arterial-venous differentiation and possible trabecular specification (Fischer et al., 2004; Koibuchi and Chin, 2007; Kokubo et al., 2007; Miao et al., 2018; Rutenberg et al., 2006; Tian et al., 2017; Xin et al., 2007; Zhong et al., 2001). In humans, HEY2 variants are associated with Brugada syndrome, a rare disease with high risk of sudden cardiac death (Bezzina et al., 2013). Although Hey proteins play pivotal roles during cardiovascular development and potential human disease, their functions in heart regeneration have not been investigated and remain unknown.

Lysine methylation is a widespread post-translational modification (Biggar and Li, 2015; Black et al., 2012). The Smyd family (Smyd1-Smyd5), containing SET and MYND domains, represents a novel class of lysine methyltransferases (KMTs) with a dual function in histone and non-histone methylation (Spellmon et al., 2015). Smyd2 methylates H3K4 to promote gene transcriptional activation, and its histone methyltransferase activity can be enhanced by interaction with HSP90α (Abu-Farha et al., 2008). By contrast, trimethylation of histone H4K20 by Smyd5 represses gene expression (Stender et al., 2012). Importantly, Smyd2 can methylate non-histone substrates during cell cycle progression, cell survival, apoptosis and other events (Gao et al., 2017; Li et al., 2017; Saddic et al., 2010).

In this study, we reveal cell-autonomous effects of Grl on regulation of myocardial regeneration using loss-of-function and conditional gain-of-function studies. Grl functions as a negative regulator by restraining injury-induced CM dedifferentiation and proliferation. We identify KMT Smyd2 upregulation at the injured myocardium as a direct target of Grl. Smyd2 induction, triggered by injury-induced Grl reduction, is required for CM proliferation during regeneration. Our findings reveal novel roles and mechanisms of the Grl-Smyd2 network in governing vertebrate CM renewal and heart regeneration.

## RESULTS

### Cardiac injury triggers a *grl* dynamic expression pattern

**Fig. 1.**
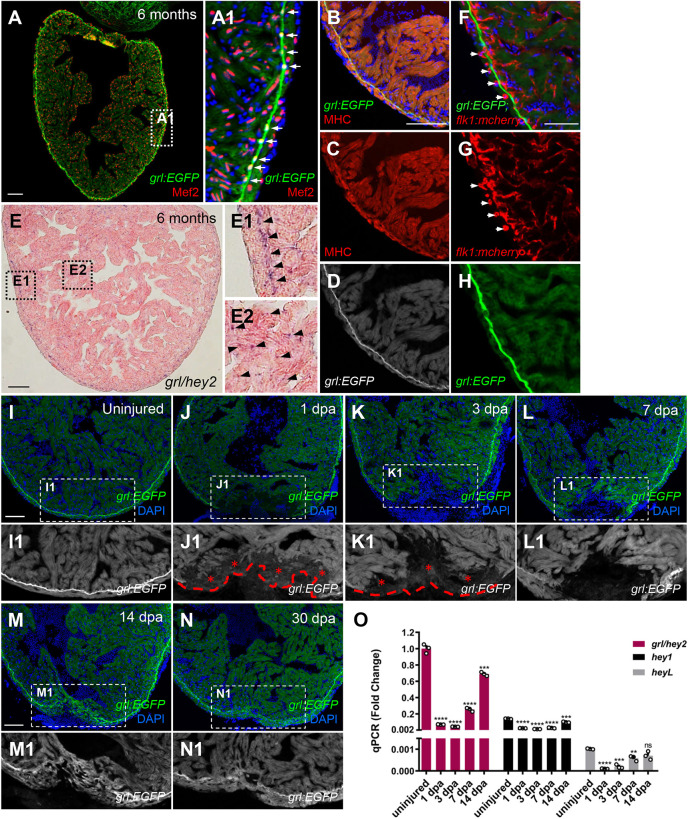
**Reduction of myocardial *grl* correlates with regenerative responses of the zebrafish heart to injury.** (A-D) *grl*:EGFP (green or white) overlaps with a nuclear CM marker Mef2 (red) (A) and a cytoplasmic CM marker MHC (red) (B-D). (A1) Enlarged image of the dashed box in A. Arrows indicate the *grl*-enriched primordial layer (PML). (E) ISH for *grl* displays enriched expression in the PML and its expression throughout the myocardium in adult zebrafish hearts. (E1) Higher-magnification image of the dashed box in E; arrowheads point to the *grl*-enriched PML. (E2) Enlarged image of the dashed box in E; arrowheads point to *grl* expression in the myocardium. (F-H) *grl*:EGFP (green) does not colocalize with endocardial or coronary endothelial cells marked by *flk1:*mCherry (red) in adult *Tg(grl:EGFP;flk1:mCherry)* hearts. Arrows indicate the circular coronary vessels. (I-N) *Tg(grl:*EGFP*)* adult heart show *grl*:EGFP (green) expression in uninjured and regenerating ventricles. (I1-N1) Higher-magnification images of the dashed boxes in I-N. Red dashed line indicates approximate plane of resection. Red asterisks mark decreased expression of *grl*:EGFP in the injury border zone. (O) Expression of *grl*, *hey1*, and *heyL* were examined using qPCR analyses in uninjured and regeneration ventricular samples. Expression levels were normalized to that of *β-actin* and further normalized to that of *grl* in uninjured sample (*n*=3). Data presents as mean±s.e.m. **P<0.01, ***P<0.001, ****P<0.0001, Student's *t*-test (unpaired, two-tailed). Scale bars: 100 µm (A-E, I-N); 50 µm (F-H).

To define the spatiotemporal expression pattern of *grl* during adult heart regeneration, we generated *grl* reporter lines *Tg(grl:EGFP)* and *Tg(grl:mCherry)* in zebrafish, where EGFP and mCherry were driven by *grl* promoter-based upstream region. In the adult heart, *grl*:EGFP expression was detectable throughout the myocardium labeled by myocyte enhance factor 2 (Mef2) and enriched in the primordial myocardial layer (PML) (Fig. 1A,A1; Fig. S1A,B). *grl*:EGFP was also overlapped with myosin heavy chain (MHC) that marked the CM cytoplasm (Fig. 1B-D). *In situ* hybridization (ISH) analyses validated *grl* expression throughout the myocardium, with enrichment in the PML (Fig. 1E). Although *grl* was expressed in the arterial endothelium of aortae during embryogenesis (Jia et al., 2007; Li et al., 2020; Rowlinson and Gering, 2010; Satow et al., 2001; Zhong et al., 2000), *grl*:EGFP was absent in the endocardium or coronary vascular endothelium lining with *flk1*:mCherry expression in the adult heart (Fig. 1F-H). Furthermore, *Tg(grl:mCherry)* transgenic hearts displayed no colocalization of mCherry with the epicardial marker *tcf21*:nucEGFP (Fig. S1C). *grl*:EGFP was also absent in the epicardium marked by injury-induced Raldh2 protein (Fig. S1D). These findings indicate that the myocardium is the primary source of Grl under homeostatic conditions.

To explore the injury response of *grl* during zebrafish heart regeneration, we performed ventricular apex resection using *Tg(grl:EGFP)* animals and investigated *grl* temporal expression profiles from injury onset until 30 days post amputation (dpa). We observed that EGFP fluorescence was reduced at the apical edge of the injured myocardium labeled by Mef2 at 1 dpa (Fig. 1J,J1; Fig. S1I,J) and declined to the lowest level at 3 dpa (Fig. 1K,K1), compared with uninjured hearts (Fig. 1I,I1). Thereafter, *grl*:EGFP increased to some extent at the injured myocardial cell edge at 7 dpa (Fig. 1L,L1;M,M1) and gradually returned to the uninjured level with formation of the PML from 14 dpa to 30 dpa (Fig. 1N,N1). Consistent with *grl*:EGFP expression, quantitative PCR (qPCR) analyses indicated a great reduction in *grl* expression at 1 dpa, which reduced to the lowest level at 3 dpa (Fig. 1O). Although *grl* expression increased to some extent at 7 dpa, it was still reduced by 75% compared with the uninjured level (Fig. 1O). Reduced expression of other Hey family genes, *hey1* and *heyL*, was also detectable by qPCR. Expression of *heyL* was much lower than that of *hey1*, despite extremely low expression of both genes (Fig. 1O). We noticed that the increased level of *grl:*EGFP transgene at 7 dpa seemed to be much higher than that in *grl* transcripts measured by qPCR (Fig. 1L,O). These differences might be because the 7.8 kb upstream region in the *grl*:EGFP transgene does not fully reflect the endogenous *grl* transcripts during regeneration. Indeed, our ISH analyses revealed a discernible increase in *grl* transcripts at the injury site at 14 dpa rather than 7 dpa (Fig. S1G,H), consistent with qPCR analyses. Collectively, our findings indicate that the reduced expression of myocardial *grl* correlates with the regenerative responses of the zebrafish heart to injury.

### Mutations in *grl* augment heart regeneration by reducing fibrotic scars and enhancing CM proliferation

**Fig. 2.**
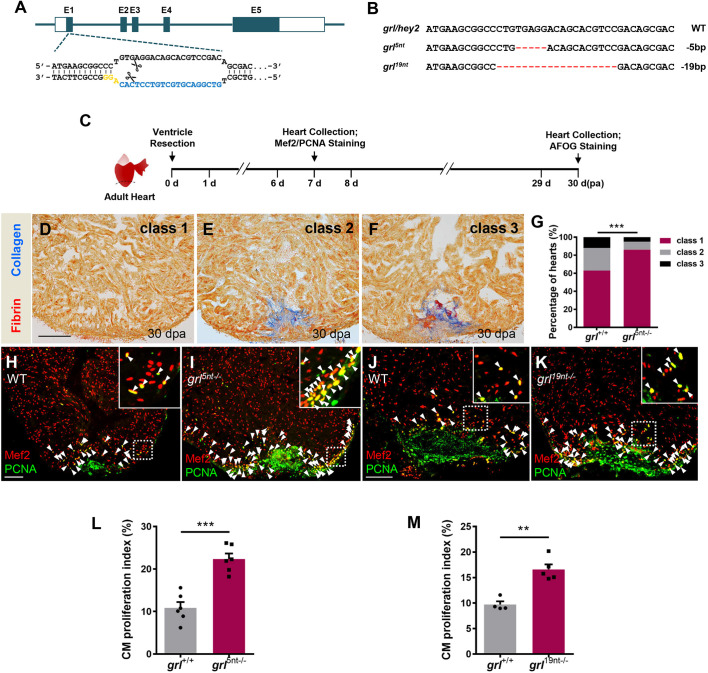
**Mutations in Grl lead to enhanced CM proliferation and reduced fibrotic scar tissue following injury.** (A) The sgRNA target sequence of the *grl* allele (blue) and the PAM (yellow) designed in the first exon of *grl* for mutation generation. (B) Targeted deletion mutations induced by CRISPR/Cas9 technique at the *grl* genes. The WT sequence is shown at the top. Deletions are shown as red dashes. The mutation deletion is indicated at the right of each sequence. (C) Experimental design for PCNA and Mef2 immunostaining and fibrotic scar (AFOG) analysis. (D-F) Representative AFOG staining images (blue for collagen, red for fibrin) of injured ventricles from WT sibling and *grl^5nt−/−^* fish at 30 dpa, scored as ‘class 1’ (complete regeneration) (D), ‘class 2’ (partial regeneration) (E) and ‘class 3’ (blockade in regeneration) (F). (G) Quantification of regenerative status of ventricles from WT sibling fish (*n*=6, sections=194) and *grl^5nt^*^−/−^ fish (*n*=8, sections=288) at 30 dpa. Heart sections were scored according to the criteria described in Materials and Methods. Histograms show the percentage of heart regeneration represented by each score for each group. ***P<0.001, Chi-square test. (H-K) Section images of injured ventricles from WT (H) and mutant *grl^5nt^*^−/−^ fish (I), as well as WT (J) and *grl^19nt−/−^* fish (K) at 7 dpa, stained with anti-PCNA (green) and anti-Mef2 (red) antibodies. Insets show higher-magnification images of the dashed boxes. Arrowheads indicate proliferating CMs. (L) Quantification of CM proliferation indices in 7 dpa ventricles of *grl*^+/+^ (*n*=6) and *grl^5nt^*^−/−^ fish (*n*=6). ***P<0.001, Student's *t*-test (unpaired, two-tailed). (M) Quantification of CM proliferation indices in 7 dpa ventricles of *grl*^+/+^ (*n*=4) and *grl^19nt^*^−/−^ fish (*n*=5). **P<0.01, Student's *t*-test (unpaired, two-tailed). Data presents as mean±s.e.m. Scale bars: 100 µm.

To investigate the effects of *grl* loss of function on heart regeneration, we generated zebrafish nonsense mutations in *grl* using the CRISPR/Cas9 technique, as a previous *grl^m145^* mutant caused a point mutation of the terminator codon that is predicted to produce an extended protein with some residue activity (Zhong et al., 2000). Single guide RNA (sgRNA) was designed to target the first exon of *grl* (Fig. 2A). Two *grl* deletion mutations, *grl^5nt−/−^* mutants with a 5-nuleotide deletion and *grl^19nt−/−^* mutants with a 19-nuleotide deletion, were identified (Fig. 2B). These mutants were predicted to produce a premature stop codon and encode a truncated peptide containing 4 amino acids in *grl^5nt−/−^* mutants or 29 amino acids in *grl^19nt−/−^* mutants, both of which lack the bHLH, Orange and YRPW domains (Fig. S2A). Some mutants survived to adulthood and were fertile. Considering that *grl^5nt−/−^* is almost a null mutation, we chose *grl^5nt−/−^* mutants to test the effects of *grl* loss of function on heart regeneration (Fig. S2B). Hearts from 5-month-old *grl^5nt−/−^* mutants were subjected to ventricular resection and then assayed for fibrin and fibrotic scar tissue using acid fuchsin-orange G (AFOG) staining at 30 dpa (Fig. 2C). We observed that more *grl^5nt−/−^* mutant hearts contained large cardiac myofiber deposits and minimal fibrin or collagen deposits than injured wild-type (WT) sibling hearts in wound regions at 30 dpa (Fig. 2D-G). Therefore, the *grl* mutant enhances cardiac muscle regeneration and reduces fibrotic scarring.

We next assessed injury-induced CM proliferation in *grl^5nt−/−^* mutant hearts by conducting immunostaining of the DNA replication marker proliferating cell nuclear antigen (PCNA) and CM marker Mef2 following ventricular resection (Fig. 2C). We observed that PCNA-positive CMs were markedly increased in *grl^5nt−/−^* hearts in comparison to injured WT sibling hearts (Fig. 2H,I). Quantification revealed that the CM proliferation index (number of PCNA^+^Mef2^+^ cells/number of Mef2^+^ cells) was significantly increased by 105% in *grl^5nt−/−^* mutant hearts compared with control hearts (Fig. 2L). Similarly, in *grl^19nt−/−^* mutants the CM proliferation index was elevated by 70% over control hearts (Fig. 2J,K,M). In contrast, the coronary vasculature was not impaired in *grl^5nt−/−^* mutant hearts (Fig. S2C,D). Collectively, these findings demonstrate that mutations in *grl* enhance injury-induced CM proliferation.

### Myocardial-specific *grl* induction in the adult heart impairs injury-induced CM dedifferentiation and proliferation

**Fig. 3.**
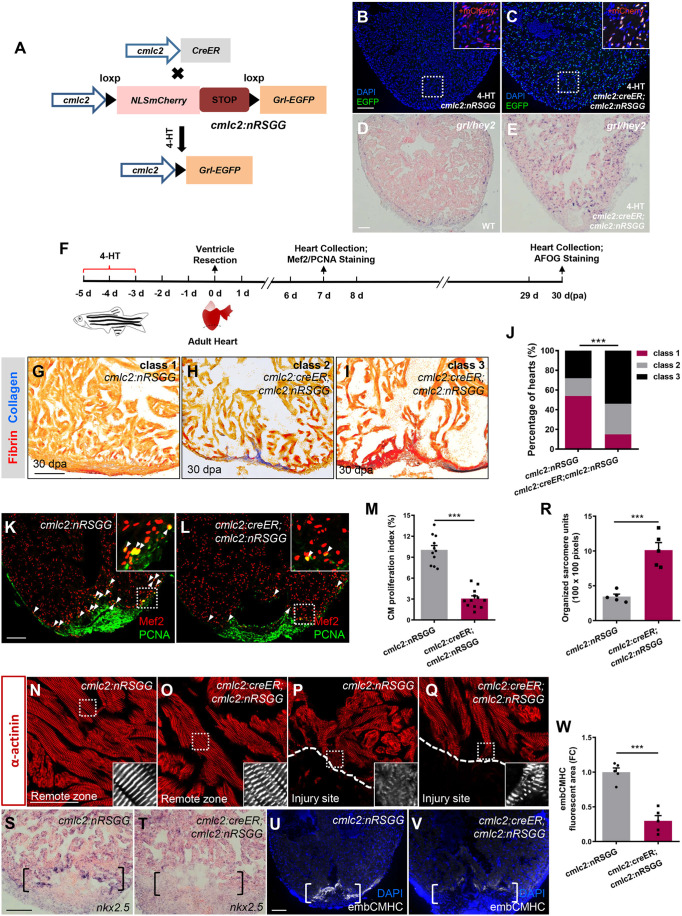
**Conditional *grl* induction in the adult myocardium impairs CM dedifferentiation and proliferation during regeneration.** (A) Transgenic zebrafish used for inducible expression of Grl-EGFP in CMs. *Tg(cmlc2:nRSGG)* zebrafish were crossed with *Tg(cmlc2:CreER)* animals, which permitted Cre-mediated recombination to induce Grl in CMs after 5 µM 4-HT treatment. (B,C) Grl was induced in CMs of 4-HT-treated *Tg(cmlc2:creER;cmlc2:nRSGG)* hearts (C) but not *Tg(cmlc2:nRSGG)* hearts (B), as indicated by EGFP protein (green). Insets show higher-magnification images of the dashed boxes adding mCherry channel (red). (D,E) ISH analyses indicate the endogenous expression of *grl* in WT hearts (D) and the induced expression of *grl* in CMs of the whole ventricle in 4-HT-treated *Tg(cmlc2:creER;cmlc2:nRSGG)* animals (E) at 7 dpa. (F) Experimental design for PCNA and Mef2 immunostaining and fibrotic scar (AFOG) analysis after ventricular resection. 4-HT treatment was 5 µM 4-HT for bath treatment for 2 days. (G-I) Section images of 30 dpa ventricles of 4-HT-treated *Tg(cmlc2:nRSGG)* control fish (G) and 4-HT-treated *Tg(cmlc2:creER;cmlc2:nRSGG)* animals (H,I) stained with AFOG (blue for collagen, red for fibrin). Heart sections were scored as ‘class 1’, ‘class 2’ or ‘class 3’ according to the criteria described in Materials and Methods. (J) Quantification of regenerative status of ventricles in 4-HT-treated *Tg(cmlc2:creER;cmlc2:nRSGG)* (*n*=6; 129 sections) and *Tg(cmlc2:nRSGG)* control group (*n*=4; 84 sections) at 30 dpa. Histograms show the percentage of wounded hearts represented by each score for each group. ***P<0.001, Chi-square test. (K,L) Immunofluorescent section images of injured ventricles from 4-HT-treated *Tg(cmlc2:nRSGG)* control fish (K) and 4-HT-treated *Tg(cmlc2:creER;cmlc2:nRSGG)* animals (L) at 7 dpa, stained with anti-PCNA (green) and anti-Mef2 (red) antibodies. Insets show higher-magnification images of the dashed boxes. Arrowheads indicate proliferating CMs. (M) Quantification of CM proliferation indices in 7 dpa ventricles; *n*=11 (*cmlc2:nRSGG*), *n*=12 (*cmlc2:creER;cmlc2:nRSGG*); ***P<0.001, Student's *t-*test (unpaired, two-tailed). (N-Q) Confocal images of sections of injured ventricles from 4-HT-treated *Tg(cmlc2:nRSGG)* control fish (N,P) and 4-HT-treated *Tg(cmlc2:creER;cmlc2:nRSGG)* animals (O,Q) at 7 dpa, stained with anti-α-actinin antibody, showing regions of the remote zone (N and O) and injury site (P and Q). Insets show higher-magnification images of the dashed boxes. Dashed line indicates the apical edge of the regeneration. (R) Quantification of organized sarcomere units in α-actinin-labeled myocardial tissue (100×100 pixels) in injury border zone of 7 dpa ventricles from 4-HT-treated *Tg(cmlc2:nRSGG)* control fish and 4-HT-treated *Tg(cmlc2:creER;cmlc2:nRSGG)* animals (*n*=5); ***P<0.001, Student's *t*-test (unpaired, two-tailed). (S,T) ISH analyses of *nkx2.5* expression in injured ventricles from 4-HT-treated *Tg(cmlc2:nRSGG)* control fish (S) and 4-HT-treated *Tg(cmlc2:creER;cmlc2:nRSGG)* animals (T) at 7 dpa (*n*=5). Brackets indicate injury site. (U,V) Fluorescent images of injured ventricles from 4-HT-treated *Tg(cmlc2:nRSGG)* control fish (U) and 4-HT-treated *Tg(cmlc2:creER;cmlc2:nRSGG)* animals (V) at 7 dpa, stained with embCMHC antibody (white). Brackets indicate injury site. (W) Quantification of embCMHC fluorescent area in injury border zone of 7 dpa ventricles from 4-HT-treated *Tg(cmlc2:nRSGG)* control fish and 4-HT-treated *Tg(cmlc2:creER;cmlc2:nRSGG)* animals. Data are relative to area in 4-HT-treated *Tg(cmlc2:nRSGG)* group (*n*=5); ***P<0.001, Student's *t*-test (unpaired, two-tailed). Data presents as mean±s.e.m. Scale bars: 100 µm (B-E,G-I,K,L,S-V); 50 µm (N-Q).

To define the potential contribution of *grl* gain of function during heart regeneration, we created a transgenic strain of zebrafish that enabled inducible expression of *grl* in CMs [*Tg(cmlc2:loxP-nlsmCherry-STOP-loxP-grl-EGFP)*, hereafter referred to as *Tg(cmlc2:nRSGG)*] (Fig. 3A). We crossed *Tg(cmlc2:nRSGG)* zebrafish with *Tg(cmlc2:CreER)* animals, in which Cre recombinase fused with estrogen receptor (ER) is under the control of the *cmlc2* promoter, permitting Cre-mediated recombination in CMs after 4-hydroxytamoxifen (4-HT) treatment (Kikuchi et al., 2010) (Fig. 3A). To test whether the inducible system works, we administrated 4-HT to *Tg(cmlc2:CreER;cmlc2:nRSGG)* and *Tg(cmlc2:nRSGG)* control animals aged 5 months. Following 4-HT treatment, Grl-EGFP signals were detectable in CM nuclei, which overlapped with nucleus-localized mCherry proteins, in *Tg(cmlc2:CreER;cmlc2:nRSGG)* hearts (Fig. 3C). In contrast, control *Tg(cmlc2:nRSGG)* hearts exhibited only mCherry nuclear signals in CMs (Fig. 3B). ISH analyses also validated abundant *grl* transcripts over the whole myocardium in *Tg(cmlc2:CreER;cmlc2:nRSGG)* hearts compared with controls (Fig. 3D,E).

Because *grl* deficiency enhances cardiac muscle regeneration, we tested whether inducible *grl* overexpression would have an opposite effect. We treated *Tg(cmlc2:CreER;cmlc2:nRSGG)* and *Tg(cmlc2:nRSGG)* control animals with 4-HT, and performed ventricular resections and fibrin and collagen assays at 30 dpa (Fig. 3F). Although the majority of 4-HT-treated control hearts largely contained cardiac muscle with minimal fibrin and fibrotic scar tissue (Fig. 3G,J), myocardial *grl* induction hearts displayed excessive fibrin and collagen deposits (Fig. 3H-J). Furthermore, CMs in *grl*-overexpressing hearts appeared to be sparsely distributed (Fig. 3H,I). We next assessed the status of CM proliferation in myocardial *grl*-overexpressing hearts following injury. Resected hearts were isolated from 4-HT-treated control and *Tg(cmlc2:CreER;cmlc2:nRSGG)* animals and assayed for PCNA and Mef2 immunostaining at 7 dpa (Fig. 3F). We observed that PCNA-positive CMs were markedly reduced in myocardial *grl*-overexpressing hearts compared with control hearts (Fig. 3K,L). The CM proliferation index was significantly decreased by 69% in *Tg(cmlc2:CreER;cmlc2:nRSGG)* hearts in comparison with control hearts (Fig. 3M). Thus, inducible *grl* overexpression in adult CMs reduces injury-induced CM proliferation.

Regenerating CMs normally undergo dedifferentiation, which is characterized by reactivation of cardiac embryonic or fetal genes as well as less organized sarcomeres in the myocardial injury region (D'Uva et al., 2015; Jopling et al., 2010; Kikuchi et al., 2010). We tested whether CM dedifferentiation might be regulated by Grl. In the region remote from injury sites, myofibrils organize in regular sarcomere units exhibiting cross-striations revealed by α-actinin in both conditional *grl*-overexpressing and control hearts (Fig. 3N,O). At the wound edge, control *Tg(cmlc2:nRSGG)* hearts exhibited disassembled cytoplasmic sarcomeres, indicative of CM dedifferentiation (Fig. 3P,R) (Jopling et al., 2010). In contrast, induced *grl*-overexpressing hearts displayed organized sarcomeres at cardiac wounds, suggesting a reduction in dedifferentiation (Fig. 3Q,R). Notably, expression of the *nkx2.5* progenitor marker and embryonic-specific cardiac myosin heavy chains (embCMHC) were markedly reduced at injury sites in myocardial *grl*-overexpressing hearts compared with control hearts at 7 dpa (Fig. 3S-W). Conversely, expression of *nkx2.5* and embCMHC were increased at wound edges in *grl* mutant hearts compared with control hearts (Fig. S3A-E). Moreover, *grl^5nt−/−^* mutant hearts exhibited conspicuously disassembled sarcomeres in comparison with control hearts (Fig. S3F-H). Collectively, these findings indicate that Grl reduces the dedifferentiation tendency of CMs during regeneration.

### Grl repressor negatively regulates transcripts encoding lysine methyltransferase Smyd2 in the regenerating myocardium

**Fig. 4.**
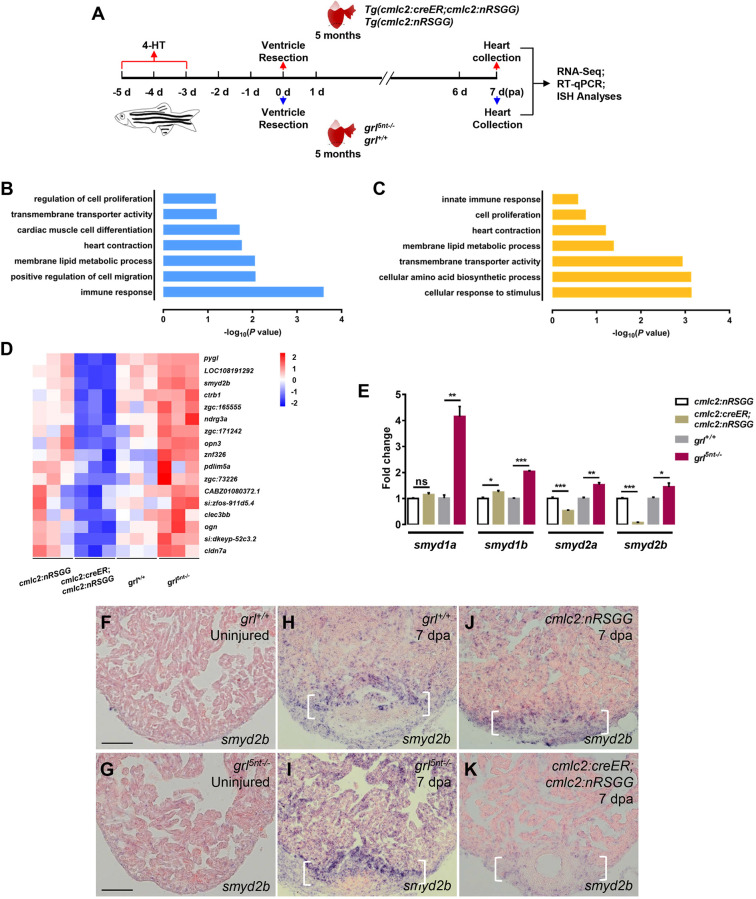
**Identification of Smyd2 methyltransferase as candidate Grl target in the regenerating heart.** (A) Experimental design for RNA-seq, RT-qPCR and ISH analyses. Red arrows represent experimental steps for *Tg(cmlc2:creER;cmlc2:nRSGG)* animals and *Tg(cmlc2:nRSGG)* control fish. Blue arrows represent experimental steps for *grl^5nt−/−^* mutant fish and WT sibling fish. (B,C) Bar graph showing −log10 *P* values for GO terms significantly represented in the downregulated gene category of the *grl*-overexpressing groups (B) and in the upregulated gene category of the *grl* mutant groups (C). (D) Heatmap indicating genes downregulated in *Tg(cmlc2:creER;cmlc2:nRSGG)* hearts and upregulated in *grl^5nt^*^−/−^ mutant hearts after resection, compared with control wounded hearts. Red, higher expression; blue, lower expression. FC>1.5, *P*<0.05. (E) qPCR analyses of *smyd1a*, *smyd1b*, *smyd2a* and *smyd2b* in injured hearts extracted from *Tg(cmlc2:creER;cmlc2:nRSGG)*, *Tg(cmlc2:nRSGG)*, *grl^5nt^*^−/−^ and *grl^+/+^* animals. Data presents as mean±s.e.m. (*n*=3); *P<0.05, **P<0.01, ***P<0.001, Student's *t*-test (unpaired, two-tailed). (F-K) ISH analyses of *smyd2b* expression in uninjured hearts of WT sibling fish (F), *grl^5nt−/−^* fish (G), and injured ventricles from WT sibling fish (H), *grl^5nt−/−^* fish (I), 4-HT-treated *Tg(cmlc2:nRSGG)* control fish (J) and 4-HT-treated *Tg(cmlc2:creER;cmlc2:nRSGG)* animals (K) at 7 dpa, respectively (*n*=5). Brackets indicate injury site. Scale bars: 100 µm.

Grl mediates transcriptional repression by binding E-box motifs (Fischer et al., 2005, 2002; Heisig et al., 2012). We reasoned that potential Grl downstream genes should be downregulated in injured hearts overexpressing *grl*, but simultaneously upregulated in *grl-*deficient hearts following damage. Therefore, we performed RNA sequencing (RNA-seq) on ventricular wound tissues collected from myocardial *grl*-overexpressing hearts, *grl^5nt−/−^* mutant hearts and their respective control groups. The 4-HT-treated *Tg(cmlc2:creER;cmlc2:nRSGG)* and *Tg(cmlc2:nRSGG)* control animals, as well as *grl^5nt−/−^* mutant and WT sibling animals, were subjected to ventricle resections. Total RNAs were isolated from injured ventricles at 7 dpa, which corresponds to when CM proliferation actively occurs (Fig. 4A), and sequenced. We generated and analyzed transcriptional profiles in *grl*-overexpressing hearts, *grl^5nt−/−^* mutant hearts and their control groups (Fig. S4A,B, Tables S1 and S2). As expected, gene ontology (GO) analyses identified categories in ‘regulation of cell proliferation’, ‘cardiac muscle cell differentiation’ and ‘heart contraction’ that were enriched in the downregulated gene set in *grl*-overexpressing RNA profiles and in the increased gene set in *grl^5nt^*^−/−^ mutant RNA profiles (Fig. 4B,C). However, we also obtained the enrichment of genes related to metabolic and biosynthetic processes, and to the immune response. We surveyed the list to identify potential Grl target genes that should be upregulated in *grl^5nt−/−^* mutant hearts and simultaneously reduced in *grl*-overexpressing hearts following injury. About 50 genes were identified as fitting the criteria and being present in overlapped RNA profile datasets (Fig. S4C, Table S2). After filtering out genes with low expression levels (FPKM<1), we obtained 17 differential expression genes containing epigenetic regulatory genes (such as *smyd2b*), metabolism regulatory genes (such as *znf326*) and other unknown factors (Fig. 4D).

We next queried these potential target genes for Grl-binding E-box content using the JASPAR database, and found that the KMT *smyd2b* contains multiple E-boxes in the regulatory upstream region (Fig. S4D). Recent studies report that Smyd family genes (*smyd1* and s*myd2*) play crucial roles in cardiomyogenesis and myofibrillogenesis (Donlin et al., 2012; Gottlieb et al., 2002; Just et al., 2011; Li et al., 2013; Tan et al., 2006; Voelkel et al., 2013). The genes *smyd1a* and *smyd1b*, *smyd2a* and *smyd2b* are duplicated in the zebrafish genome. Similar to *smyd2b*, the regulatory upstream region in *smyd2a* and human *SMYD2* contains E-box motifs (Fig. S4E,F). Quantitative PCR analyses indicated that expression of *smyd1a* and *smyd1b*, *smyd2a* and *smyd2b* were upregulated in wounded *grl^5nt−/−^* mutant hearts (Fig. 4E). However, expression of *smyd2a* and *smyd2b* but not *smyd1a* and *smyd1b* were reduced in 4-HT-treated *grl*-overexpressing hearts following injury (Fig. 4E), suggesting that expression of *smyd2a* and *smyd2b* is transcriptionally repressed by Grl during cardiac regeneration.

To assess whether *smyd2* expression is normally upregulated by injury, we analyzed and compared *smyd2b* expression in uninjured and amputated WT hearts, considering *smyd2a* expression is very low (data not shown). Using ISH analyses, we observed a marked induction of *smyd2b* expression at the wound edge and a slight increase in the remote myocardial area in WT hearts at 7 dpa (Fig. 4H), whereas *smyd2b* transcripts were hardly detectable in uninjured WT hearts (Fig. 4F). Next, we determined whether *smyd2* induction was controlled by Grl during regeneration. We noticed that *grl^5nt−/−^* mutant hearts following resection displayed much stronger *smyd2b* upregulation at wound edges and remote areas compared with that in injured WT sibling hearts (Fig. 4I,H). Similar to uninjured WT hearts, *smdy2b* transcripts were hardly detectable in unresected *grl^5nt−/−^* mutant hearts (Fig. 4G), suggesting that *grl* reduction is not sufficient to induce *smyd2b* expression in uninjured hearts. Remarkably, myocardial-specific *grl* overexpression abolished *smyd2b* induction at the wound region and remote areas, compared with that in injured *Tg(cmlc2:nRSGG)* control hearts (Fig. 4J,K). Collectively, these findings demonstrate that Grl negatively regulates *smyd2b* expression throughout the myocardium in response to injury.

### Smyd2, acting as a key Grl transcriptional target, is required for heart regeneration by enhancing CM proliferation

**Fig. 5.**
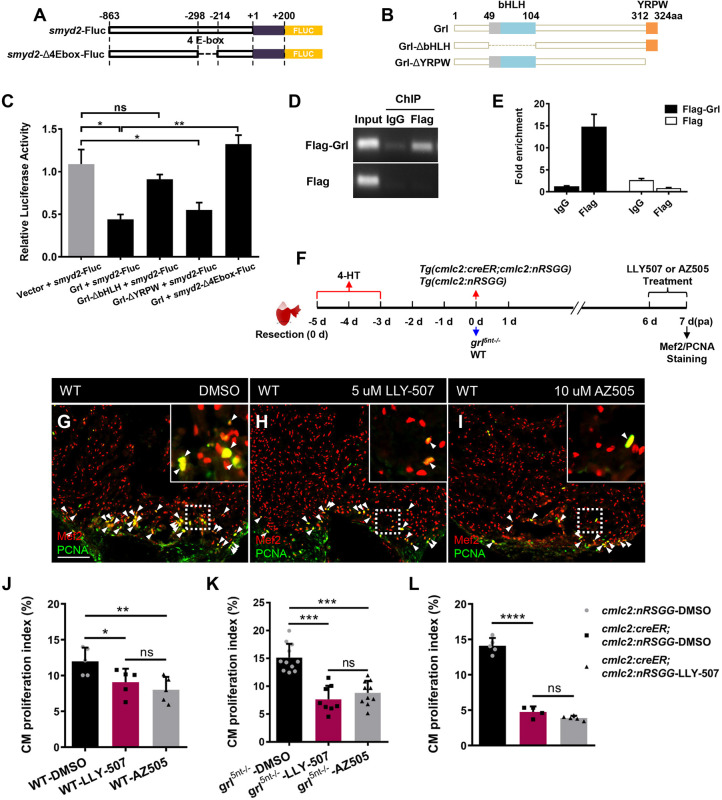
**Smyd2 acts as a transcriptional target of Grl to promote heart regeneration.** (A) E-box deletion in the *smyd2* promoter for luciferase assays. (B) Deletion (Δ) fragments of Grl created for luciferase assays. (C) Luciferase activity in cells after co-transfection of pGL3-*smyd2*-Fluc with control plasmid vector, Grl, Grl-ΔbHLH or Grl-ΔYRPW expression plasmids, as well as transfection of pGL3-*smyd2*-Δ4Ebox-Fluc with Grl expression plasmids. The relative flyfire luciferase activity was normalized by *Renilla* luciferase and calculated as the ratio of each experimental group to the control group (*n*=3). (D,E) ChIP-PCR (D) and ChIP-qPCR (E) analyses of enrichment of Grl at its predicted binding sites in *smyd2* promoter in HEK 293T cell. Enrichment levels of *smyd2* promoter fragment were examined in pEGFP-Flag-grl and pEGFP-Flag transfected groups immunoprecipitated with anti-IgG or anti-Flag antibody. Genomic DNA isolated before IP was analyzed as the input control. Enrichment levels of qPCR analysis were normalized to that in the pEGFP-Flag-grl transfected group immunoprecipitated with IgG. (F) Experimental design for inhibitor treatment and CM proliferation analyses after ventricular resection in WT, *grl* mutant hearts, as well as in 4-HT-treated *Tg(cmlc2;CreER;cmlc2:nRSGG)* hearts and 4-HT-treated *Tg(cmlc2;nRSGG)* control hearts. Treatment at 6-7 dpa was with 5 µM LLY-507, 10 µM AZ505 or 0.5‰ DMSO. Red arrows represent experimental steps for *Tg(cmlc2:creER;cmlc2:nRSGG)* animals and *Tg(cmlc2:nRSGG)* control fish. Blue arrows represent experimental steps for *grl^5nt−/−^* mutant fish and WT sibling fish. (G-I) Immunofluorescent section images of injured ventricles from DMSO- (G), LLY-507- (H) and AZ505-treated (I) WT fish at 7 dpa, stained with anti-PCNA (green) and anti-Mef2 (red) antibodies. Insets show higher-magnification images of the dashed boxes. Arrowheads indicate proliferating CMs. (J) Quantification of CM proliferation indices in 7 dpa ventricles derived from DMSO-, LLY-507- or AZ505-treated WT fish (*n*=5). (K) Quantification of CM proliferation indices in 7 dpa ventricles derived from DMSO- (*n*=11), LLY-507- (*n*=8) or AZ505-treated *grl^5nt^*^−/−^ fish (*n*=10). (L) Quantification of CM proliferation indices in 7 dpa ventricles derived from DMSO-treated *Tg(cmlc2:nRSGG)* control fish (*n*=5), DMSO-treated *Tg(cmlc2:creER;cmlc2:nRSGG)* fish (*n*=4) and LLY-507-treated *Tg(cmlc2:creER;cmlc2:nRSGG)* fish (*n*=5). Data presents as mean±s.e.m. *P<0.05, **P<0.01, ***P<0.001, ****P<0.0001, Student's *t*-test (unpaired, two-tailed). Scale bars: 100 µm.

To assess whether Grl directly mediates transcriptional repression of *smyd2*, we performed luciferase reporter assays in HEK 293T cells. We generated a pGL3-*smyd2*-Fluc vector that links the *smyd2* promoter containing four E-box motifs with firefly luciferase (Fluc) (Fig. 5A), as well as a pGL3-*smyd2-Δ4Ebox*-Fluc plasmid containing the Fluc-fused *smyd2* promoter deleting four E-boxes (Fig. 5A). Grl-truncated mutants harboring deletions of the bHLH domain (Grl-ΔbHLH) and the YRPW motif (Grl-ΔYRPW) were also constructed (Fig. 5B). We co-transfected dual luciferase expression plasmid pGL3-*smyd2*-Fluc with pcDNA3-Grl, pcDNA3-Grl-ΔbHLH or pcDNA3-Grl-ΔYRPW. Grl transfection efficiently reduced *smyd2* luciferase activity by 60% in comparison with pcDNA3 controls (Fig. 5C). By contrast, Grl-ΔbHLH mutant failed to reduce *smyd2*-luciferase activity, whereas the Grl-ΔYRPW mutant maintained the repression of *smyd2*-luciferase activity (Fig. 5C), indicating that Grl represses *smyd2* transcription via its bHLH domain. Remarkably, deletion of four E-box motifs in the *smyd2* promoter abrogated repression responsiveness to Grl when pcDNA3-Grl and pGL3-*smyd2*-*Δ4Ebox*-Fluc were co-transfected (Fig. 5C). To assess whether Grl binds the *smyd2* promoter region, chromatin-immunoprecipitation (ChIP) was conducted by transfection of pEGFP-Flag-Grl or pEGFP-Flag control plasmids into cells. ChIP-PCR analyses revealed enrichment of the E-box-containing promoter region of *smyd2* in the chromatin-immunoprecipitates using anti-Flag antibody, whereas the control IgG antibody did not enrich *smyd2* promoter region (Fig. 5D,E). Whole genome ChIP-seq analyses for human HEY2 also identified enrichment of the *SMYD2* promoter region (Heisig et al., 2012). Collectively, these results demonstrate that Grl mediates transcriptional repression of *smyd2* by binding to the E-box-containing promoter, indicating that *smyd2* is a direct transcriptional target of Grl, consistent with RNA profiling analyses in the setting of heart regeneration.

Because Smyd2 is induced mainly in the myocardium following injury, we tested whether Smyd2 functions as an essential regulator to promote CM proliferation during regeneration. LLY-507 and AZ505 are chemical inhibitors that specifically block the KMT activity of Smyd2 (Gao et al., 2017; Li et al., 2017; Nguyen et al., 2015). We administrated resected WT animals with LLY-507 or AZ505, and performed immunostaining with PCNA and Mef2 (Fig. 5F). Inhibition of Smyd2 KMT activity by LLY-507 treatment caused a marked reduction in CM proliferation, leading to a 25% reduction in the CM proliferation index in comparison with vehicle treatment (Fig. 5G,H,J). Similarly, administration of injured WT hearts with AZ505 inhibitor reduced the CM proliferation index by 34% (Fig. 5G,I,J). These findings indicate that the KMT activity of Smyd2 is required for CM proliferation during regeneration. Next, we assessed whether the elevated CM proliferation in *grl* mutant hearts might be due to Smyd2 induction following damage. We administrated *grl^5nt−/−^* mutant animals with LLY-507 or AZ505 treatment following resections and analyzed the CM proliferation indices (Fig. 5F). Inactivation of Smyd2 activity with LLY-507 or AZ505 treatment resulted a significant reduction in the CM proliferation indices in *grl^5nt−/−^* hearts compared with vehicle-treated *grl^5nt−/−^* hearts (Fig. 5K; Fig. S5A-C). Considering that Smyd2 is a transcriptional repression target of Grl, we assessed CM proliferation in myocardial *grl* induction hearts with Smyd2 inactivation (Fig. 5F). LLY-507-treated *grl* induction hearts displayed almost the same percentage reduction in CM proliferation indices as in vehicle-treated hearts following resection (Fig. 5L; Fig. S5D-F), suggesting that *smyd2* functions in the *grl* pathway during regeneration.

### Smyd2 lysine methyltransferase mediates Stat3 activation during heart regeneration

**Fig. 6.**
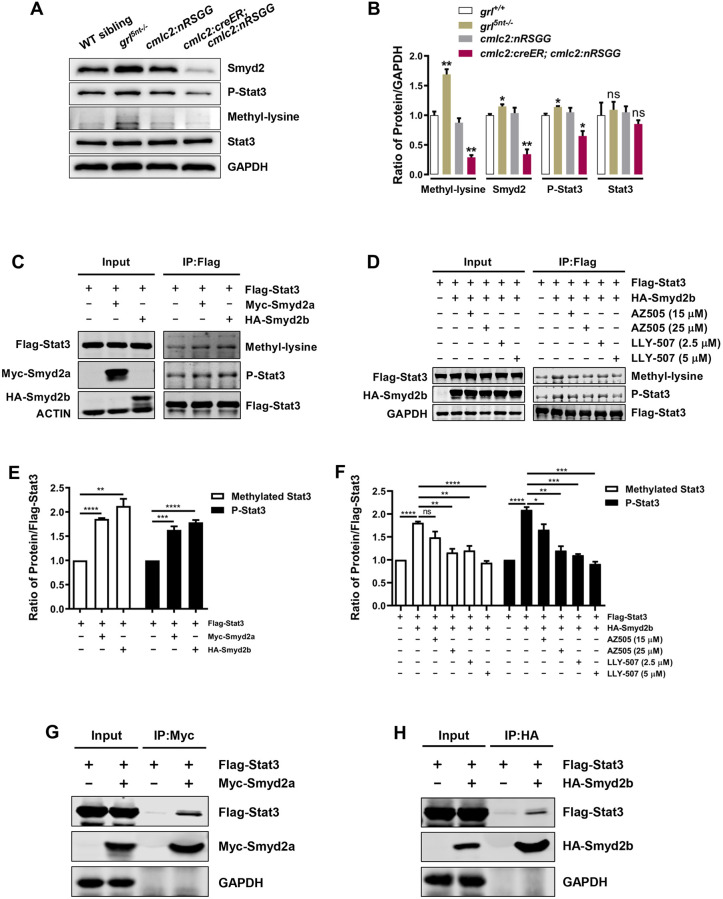
**Smyd2 functions as a KMT and controls Stat3 methylation and phosphorylation.** (A) western blot analysis exhibiting Smyd2, Stat3, P-Stat3 or corresponding lysine methylated proteins using total lysates extracted from WT sibling, *grl^5nt−/−^*, 4-HT-treated control *Tg(cmlc2:nRSGG)* hearts or 4-HT-treated *Tg(cmlc2:creER;cmlc2:nRSGG)* hearts at 7 dpa, respectively. GAPDH was used as a loading control. (B) Quantification of western blots using ImageJ software and normalized to GAPDH (*n*=3). (C) Methylation and phosphorylation assay showing increased levels of methylated Stat3 and P-Stat3 in Flag-Stat3 and Myc-Smyd2a/HA-Smyd2b co-transfected HEK 293T cells. The cell lysates were used to immunoprecipitate (IP) Stat3 with anti-Flag antibody and then blotted with anti-methyl-lysine or anti-P-Stat3 antibodies. (D) Treatment with AZ505 or LLY-507 diminishes Stat3 methylation and phosphorylation in cells co-transfected with Flag-Stat3 and HA-Smyd2b. The cell lysates were treated with AZ505 (15 µM for 6 h, 25 µM for 6 h) or LLY-507 (2.5 µM for 28 h, 5 µM for 28 h), used to IP Stat3 using anti-Flag antibody and then blotted with anti-methyl-lysine and anti-P-Stat3 antibodies. (E) Quantification of methylated Stat3 and phosphorylated Stat3 levels normalized to Flag-Stat3 (*n*=3). (F) Quantification of methylated Stat3 and phosphorylated Stat3 levels normalized to Flag-Stat3 in cells treated with AZ505 or LLY-507 (*n*=3). (G,H) Smyd2a or Smyd2b immunoprecipitation with anti-Myc antibody (G) or anti-HA antibody (H), respectively, detects Stat3 using anti-Flag antibody. HEK 293T cells were co-transfected with constructs of Flag-Stat3 and Myc-Smyd2a (G) or HA-Smyd2b (H). GAPDH was used as a loading control. Data represents mean±s.e.m. *P<0.05, **P<0.01, ***P<0.001, ****P<0.0001, Student's *t*-test (unpaired, two-tailed).

As a KMT, Smyd2 conducts its function by methylation of histones or non-histone substrates, including Stat3, during cell proliferation, survival and other events (Donlin et al., 2012; Gao et al., 2017; Li et al., 2017). We first assessed whether Smyd2 regulates histone methylation during Grl-mediated heart regeneration. Methylation of H3K4 and H3K9 were examined using cardiac wound tissues extracted from *grl^5nt−/−^* mutant hearts or *grl*-overexpressing hearts. We learned that various methylation patterns of H3K4 and H3K9 appeared not to be affected in *grl* loss- or gain-of-function hearts (Fig. S6). On the contrary, levels of phosphorylated Stat3 (P-Stat3) and Smyd2 were both increased or reduced in *grl^5nt−/−^* mutant hearts or *grl*-overexpressing hearts, respectively, whereas Stat3 levels failed to be affected (Fig. 6A,B), revealing correlation of the phosphorylated Stat3 level with the Smyd2 level during regeneration. Previous studies report that, during renal cystic tissue growth, Stat3 phosphorylation is induced by Smyd2 through methylation of its own protein (Li et al., 2017, 2018a), leading to increased renal epithelial cell proliferation. Phosphorylated active Stat3 is also mitogenic and required for heart regeneration in zebrafish (Fang et al., 2013). We hypothesized that Smyd2 regulates myocardial regeneration by modulating the Stat3 methylation and phosphorylation activity. Anti-methylated lysine (methyl-lysine) antibody, a pan-methyl antibody available in the field, was used to recognize lysine-methylated proteins. We found that methylated proteins corresponding to the size of Stat3 were correlated with the P-Stat3 level or the Smyd2 level in *grl^5nt−/−^* mutant hearts or *grl*-overexpressing hearts, respectively (Fig. 6A,B). We tested whether zebrafish Smyd2a or Smyd2b directly regulates Stat3 methylation and phosphorylation. Plasmids containing pcDNA3-Flag-Stat3 and pcDNA3-Myc-Smyd2a or pcDNA3-HA-Smyd2b were co-transfected into HEK 293T cells. We found that coexpression of Stat3 with Smyd2a or Smyd2b in HEK 293T cells resulted in an increase in levels of both Stat3 methylation and phosphorylation (Fig. 6C,E). In contrast, inactivating Smyd2 KMT activity with inhibitor AZ505 or LLY-507 not only caused a reduction in methylated Stat3, but also led to a decrease in phosphorylated Stat3 (Fig. 6D,F). Furthermore, co-immunoprecipitation (Co-IP) analyses revealed physical interactions between Flag-Stat3 with Myc-Smyd2a or HA-Smyd2b (Fig. 6G,H). Together, these findings indicate that zebrafish Smyd2 interacts with Stat3 to regulate the methylation and phosphorylation of Stat3 in cultured cells, in agreement with previous murine studies (Li et al., 2018a).

**Fig. 7.**
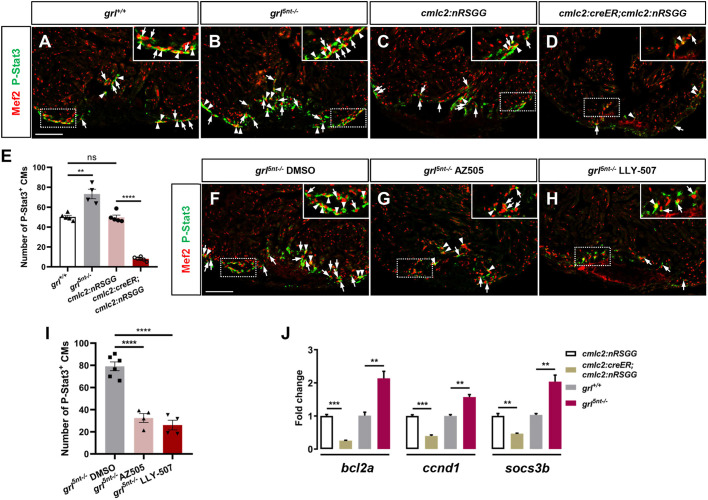
**Grl-Smyd2 mediates Stat3 activation during heart regeneration.** (A-D) Immunofluorescent images of injured ventricles stained with anti-P-Stat3 antibody (green) and anti-Mef2 antibody (red) from WT siblings (A), *grl^5nt−/−^* mutant fish (B), 4-HT-treated *Tg(cmlc2:nRSGG)* control animals (C) and 4-HT-treated *Tg(cmlc2:creER;cmlc2:nRGG)* animals (D) at 7 dpa. Insets show higher-magnification images of the dashed boxes. Arrowheads indicate P-Stat3 overlapping with Mef2 in CM nuclei; Arrows indicate perinuclear cytosolic P-Stat3 around Mef2 nuclei. P-Stat3 immunostaining was also detectable in non-CM cells in the injured area. (E) Quantification of number of P-Stat3-positive CMs in the injury border zone of 7 dpa ventricles from WT siblings (*n*=5), *grl^5nt−/−^* mutants (*n*=4), *Tg(cmlc2:nRSGG)* control fish (*n*=5), or *Tg(cmlc2:creER;cmlc2:nRGG)* fish (*n*=6). (F-H) Immunofluorescent section images of injured ventricles stained with anti-P-Stat3 antibody (green) and anti-Mef2 antibody (red) from DMSO-treated (F), AZ505-treated (G) and LLY-507-treated *grl^5nt^*^−/−^ mutant fish (H) at 7 dpa. Insets show higher-magnification images of the dashed boxes. Arrowheads indicate P-Stat3 overlapping with Mef2; Arrows indicate perinuclear cytosolic P-Stat3 around Mef2 nuclei. (I) Quantification of number of P-Stat3-positive CMs in injury border zones at 7 dpa from DMSO-treated (*n*=6), AZ505-treated (*n*=4) and LLY-507-treated *grl^5nt^*^−/−^ mutant fish (*n*=4). (J) qPCR analyses of *bcl2a*, *ccnd1* and *socs3b* in injured hearts extracted from *Tg(cmlc2:creER;cmlc2:nRSGG)* and *Tg(cmlc2:nRSGG)* control fish, as well as WT sibling and *grl^5nt^*^−/−^ mutant fish (*n*=3). Data presents as mean±s.e.m. **P<0.01, ***P<0.001, ****P<0.0001, Student's *t*-test (unpaired, two-tailed). Scale bars: 100 µm.

Next, we assessed whether Grl-Smyd2 modulates Stat3 methylation and phosphorylation activity during heart regeneration. Following injury, injured hearts derived from *grl* mutants and WT siblings, as well as *grl*-overexpressing and control animals, were subjected to double immunostaining with anti-P-Stat3 and anti-Mef2 antibodies (Fig. 7A-D). Previous studies report that P-Stat3 is primarily detectable in the nucleus, but certain human tissues and cells also display cytosolic and perinuclear P-Stat3, some of which goes to the nuclei afterwards (Chen et al., 2006; Lee et al., 2017; Li et al., 2018b; Zhang et al., 2019). During heart regeneration, we observed that P-Stat3 partially overlapped with Mef2 in CM nuclei in the injury region, whereas some P-Stat3 localized in the cytoplasmic area closely around CM nuclei (Fig. 7A). P-Stat3 was also detected in non-CM cells at the wound region (Fig. 7A). However, P-Stat3 was hardly detectable in CMs or non-CM cells in the remote area of injured hearts (Fig. 7A), suggesting that the murine P-Stat3 antibody recognizes proliferative CMs at the injury region in zebrafish hearts, despite some nonspecific signals. We distinguished and quantified P-Stat3-positive CMs in the injury region, including CMs with nuclear P-Stat3 labeling and perinuclear cytosolic P-Stat3 staining (Fig. 7A-I). Notably, P-Stat3-positive CMs were increased at injury regions in *grl^5nt−/−^* mutant hearts compared with WT sibling hearts (Fig. 7A,B,E). Inversely, myocardial *grl* induction caused a marked reduction in P-Stat3-labeled CMs at wound areas in comparison with *Tg(cmlc2:nRSGG)* control hearts (Fig. 7C-E), indicating the negative regulation of P-Stat3-labeled CMs by Grl at the cardiac wound. To establish connections between the methyltransferase activity of Smyd2 and Stat3 phosphorylation, we treated injured *grl^5nt−/−^* mutant hearts with inhibitors AZ505 and LLY-507. Consistent with our hypothesis, inhibiting Smyd2 KMT activity with AZ505 or LLY507 following injury reduced P-Stat3-labeled CMs in *grl^5nt−/−^* mutant hearts compared with vehicle-treated *grl^5nt−/−^* hearts (Fig. 7F-I), suggesting the contribution of Smyd2 KMT activity to the increased P-Stat3-labeled CMs in *grl* mutant hearts. Furthermore, expressions of *B-cell lymphoma 2* (*bcl2a*), *cyclin D1* (*ccnd1*) and *suppressor of cytokine signaling 3b* (*socs3b*), target genes in the Stat3 pathway, were reduced in *grl*-overexpressing hearts and increased in *grl* mutant hearts (Fig. 7J). Taken together, these findings suggest that the effects of Grl on heart regeneration are mediated at least in part by Smyd2-dependent Stat3 activation.

## DISCUSSION

In this study, we describe the transcriptional control of methylation modulation in governing CM renewal and heart regeneration. We demonstrate that Grl functions as a negative regulator to restrain heart regeneration in zebrafish, and identify KMT Smyd2 as the Grl-repressing target in this context. Following cardiac injury, Grl reduction triggers Smyd2 induction at the injured myocardial cell edge, leading to CM proliferation and heart regeneration. We show that zebrafish Smyd2 functions as a KMT and regulates CM renewal in part by modulating the activity of Stat3. These findings suggest that manipulation of the Grl-Smyd2 network might be instrumental in potential strategies to relieve cardiac barriers and accelerate heart regeneration in mammalian hearts.

The limited regenerative potential of adult mammalian hearts can be attributed to cell-intrinsic barriers that prevent CMs from entering the cell cycle (González-Rosa et al., 2018; Li et al., 2015; Tzahor and Poss, 2017). Recent studies have uncovered a few molecular blocks that impede zebrafish heart regeneration (Dogra et al., 2017; Jopling et al., 2012; Missinato et al., 2018). We demonstrate that Grl acts as a transcriptional repressor to limit injury-induced CM proliferation. This is consistent with its roles in negative regulation of embryonic heart development and growth in zebrafish (Jia et al., 2007). Hey family genes play multiple roles during embryogenesis, with expression in various cell types (Fischer et al., 2002; Nakagawa et al., 1999; Zhong et al., 2000). In the embryonic heart, *grl* is enriched in the myocardial compact zone, as well as weakly expressed in the trabecular region and ventricular endocardium (Jia et al., 2007; Miao et al., 2018). During murine heart development, *hey2* expression in myocardial compact zones and ventricular endocardium is regulated by Notch signaling (Miao et al., 2018), but its functional mechanisms remain unknown. In the adult zebrafish heart, we observed that *grl* expression is restricted to myocardial cells but not the endocardium or coronary vasculature. Notably, myocardial *grl* expression in the adult heart is reduced in response to heart damage. By contrast, Notch signaling is upregulated in endocardial and epicardial cells following injury, stimulating CM renewal in zebrafish (Zhao et al., 2019). Thus, the role of myocardial Grl in limiting heart regeneration is independent of endocardial Notch signaling. We observed that *grl* expression declines to the lowest level from 1 dpa to 3 dpa, increases to some extent at 7 dpa, and gradually restores from 14 dpa to 30 dpa. During regeneration, many factors that stimulate CM proliferation are induced after injury, which can counteract inhibitory factors such as Grl for CM proliferation. We believe that the spatiotemporal regulation of genome-wide networks enables CMs to regenerate after injury. Overall, the temporal expression patterns of *grl* are consistent with the functional model in which Grl negatively regulates CM proliferation during zebrafish heart regeneration. It is important to test whether murine *Hey2* expression is reduced following cardiac injury, and whether conditional *Hey2* deletion in mice can promote CM regeneration.

Smyd2 represents a novel class of KMTs and has been reported to methylate histone and non-histone substrates (Donlin et al., 2012; Gao et al., 2017; Li et al., 2017). Our studies identify a crucial role for Smyd2 during heart regeneration. However, Smyd2 is dispensable for cardiac development in mice (Diehl et al., 2010), suggesting that Smyd2 is required for injury-induced heart regeneration or functions in response to other cardiac stresses, consistent with our observations that zebrafish *smyd2* expression was not detectable in uninjured adult hearts. In addition to Stat3, Smyd2 methylates other non-histone substrates, including BMP receptor 2 (BMPR2), NF-κB and HSP90 (Donlin et al., 2012; Gao et al., 2017; Li et al., 2017). It is likely that Smyd2 has multiple methylation substrates during cardiac regeneration. It is important to assess whether Smyd2 regulates methylation of NF-κB or BMPR2 following injury. Alternatively, Smyd2 might methylate chaperone Hsp90 to promote interaction of the methyl-Hsp90-sarcomeric protein complex and myofilament organization during heart regeneration (Donlin et al., 2012). Future studies are warranted to investigate various methylation substrates of Smyd2 during cardiac regeneration, and to assess the extent to which methylation modulations play crucial roles during Grl-mediated transcriptional controls. The findings of such studies will reveal novel transcriptional regulations and various methylation controls that direct vertebrate heart regeneration, which will be relevant in developing strategies for regeneration interventions in humans.

## MATERIALS AND METHODS

### Zebrafish strains

WT zebrafish of the AB strain were used for generating *grl* mutants and various transgenic lines. Published transgenic lines used in this study included *Tg(cmlc2:mCherry)* (Palencia-Desai et al., 2011), *Tg(flk1:EGFP)* (Jin et al., 2005), *Tg(flk1:mCherry)* (Ren et al., 2013), *Tg(tcf21:nucEGFP)* (Wang et al., 2011) and *Tg(cmlc2:CreER)* (Kikuchi et al., 2010). The Institutional Animal Care and Use Committee at East China Normal University advises animal care and research.

### Generation of *grl* mutants

The *grl* mutants were generated using CRISPR/Cas9-mediated mutagenesis. *Cas9* capped mRNA was produced by *in vitro* transcription from a pGH-T7-zCas9 vector (Liu et al., 2014) using mMESSAGE mMACHINE T7 Transcription Kit (Ambion). The sgRNA target site of *grl* was designed via the CRISPRscan website. The sgRNA transcription template was PCR amplified and then transcribed using MAXIscript T7 Kit (Ambion). *Cas9* capped mRNA and sgRNA were co-injected into zebrafish embryos at the one-cell stage. The final concentrations of *Cas9* mRNA and sgRNA were 400 ng/µl and 30 ng/µl, respectively.

### Generation of *Tg(grl:EGFP)*, *Tg(grl:mCherry)* and *Tg(cmlc2:loxP-nlsmCherry-STOP-loxP-grl-EGFP)* zebrafish

To generate *Tg(grl:EGFP)* zebrafish, a 7.8 kb fragment containing the *grl* promoter sequence was amplified from BAC14 (Zhong et al., 2000) and cloned into the pCR-Blunt II-TOPO vector. A 2888 bp *tol2 3′end*-*tol2 5′end*-*EGFP* cassette was amplified from pT2KXIGΔin vector (Urasaki et al., 2006), discarding the original EF1p promoter. After verification of the *grl* promoter sequence, a 7.6 kb *grl* promoter fragment flanked by a 24-nucleotide overlap of *tol2 3′end*-*tol2 5′end*-*EGFP* cassette was amplified and assembled with this cassette using Gibson Assembly Master Mix (NEB). DNA construct (40 ng/µl) and Tol2 transposase mRNA (50 ng/µl) prepared from pCS-TP were co-injected into zebrafish embryos at the one-cell stage. To generate the *Tg(grl:mCherry)* line, the *EGFP* sequence in pT2KXIGΔin vector was replaced with the *mCherry* fragment. The methods used were the same as described above.

To generate the *Tg(cmlc2:nRSGG)* strain, full name as *Tg(cmlc2:loxP-nlsmCherry-STOP-loxP-grl-EGFP)*, the MultiSite Gateway Kit (Invitrogen) was used to simultaneously clone a *cmlc2*:*loxP-nlsmCherry-STOP-loxP* cassette, *grl* CDS fragment and *EGFP* fragment into the pDESTol2pA2 destination vector. The *cmlc2*:*loxp-nlsmCherry-STOP-loxp* cassette was flanked by *attB4* and *attB1r*. The *grl* CDS fragment was flanked by *attB1* and *attB2*. The *EGFP* fragment was flanked by *attB2r* and *attB3*. Three *attP*-containing donor vectors (pDONR P4-P1R, pDONR 221 and pDONR P2R-P3) were used in separate BP recombination reactions with the above-mentioned *attB*-containing PCR fragments to generate three entry clones. The three entry clones and the pDESTol2pA2 destination vector were used together in an LR recombination reaction between *attL*- and *attR*-flanked regions to create the *cmlc2:loxP-nlsmCherry-STOP-loxP-grl-EGFP* construct. The construct was co-injected with Tol2 transposase mRNA into one-cell stage embryos to generate transgenic lines, which were screened for nlsmCherry and then EGFP upon Cre-mediated recombination.

### Ventricular apex resection and chemical treatment

Zebrafish at 5-6 months of age were used for ventricular resection surgery as previously described (Poss et al., 2002). About 20% of ventricular muscle was excised from the ventricle apex after anesthesia as previously described (Poss et al., 2002). 4-HT (Sigma) was dissolved in ethanol to give a 20 mM stock solution. To induce expression of *grl* in adult zebrafish heart, *Tg(cmlc2:creER;cmlc2:nRSGG)* fish were incubated with 5 µM 4-HT for bath treatment for 2 days. After 24 hours, fresh 4-HT was prepared and added for an additional 24 h treatment. AZ505 ditrifluoroacetate (MCE) was dissolved in DMSO to give a 20 mM stock solution and diluted in aquarium water to 10 µM for bath treatment from 6 to 7 dpa. LLY-507 (MCE) was dissolved in DMSO to give a 10 mM stock solution and diluted in aquarium water to 5 µM for bath treatment from 6 to 7 dpa. DMSO (0.5‰) was used as a vehicle control for AZ505 and LLY-507 treatment.

### Fibrin and fibrotic scar analysis, *in situ* hybridization and immunofluorescence

Zebrafish hearts were fixed in 4% paraformaldehyde (PFA) at room temperature for 1 h. All histological experiments were performed using 10 µm cryosections. Acid fuchsin-orange G (AFOG) staining for fibrin and fibrotic scar analyses was performed as described (Poss et al., 2002). ISH was performed using digoxigenin-labeled RNA antisense probes as described (Lepilina et al., 2006). Probes were used at a concentration of 2 ng/µl diluted with hybridization buffer. The hybridization was performed overnight at 65°C. Detection was done using 1:5000 dilution of anti-digoxigenin-AP (Roche) and visualized by NBT/BCIP (Roche) substrate reacting at 37°C. Immunofluorescence was performed as described (Kikuchi et al., 2011). Cryosections were administrated with antigen retrieval at 98°C for 20 min (for PCNA staining), washed three times in PBS containing 0.1% Tween-20 and incubated in blocking buffer (3% BSA, 5% normal sheep serum, 1% DMSO, 0.1% Triton-X100, 0.1% Tween-20 in PBS) at 37°C for 30 min. The sections were incubated with primary antibody overnight at 4°C. After washing, the sections were incubated with secondary antibody for 2 h at 37°C and then mounted with Vectashield mounting medium (Vector laboratories) for imaging. Primary antibodies used in this study were as follows: anti-mCherry (Invitrogen; M11217; 1:200), anti-Mef2 (Santa Cruz; sc-313; 1:100), anti-MHC (DSHB; MF20; 1:200), anti-PCNA (Sigma; P8825; 1:200), anti-Raldh2 (ABclonal; A7503; 1:200), anti-α-actinin (Abcam; ab9465; 1:100), anti-α-actinin (Abcam; ab68167; 1:100), embCMHC antibody (DSHB; N2.261; 1:100) and anti-P-Stat3 (Santa Cruz; sc-8059; 1:100). Secondary antibodies used in this study were as follows: Alexa Fluor 555 goat anti-rat IgG (H+L) (Invitrogen; A-21434; 1:800), Alexa Fluor 594 goat anti-rabbit IgG (H+L) (Invitrogen; A-11012; 1:800), Alexa Fluor 488 goat anti-mouse IgG (H+L) (Invitrogen; A-11001; 1:800), Alexa Fluor 594 goat anti-mouse IgG (H+L) (Invitrogen; A-11005; 1:800) and Alexa Fluor 647 goat anti-mouse IgG (H+L) (Invitrogen; A-21235; 1:800).

To quantify regenerative status shown by AFOG staining at 30 dpa, we scored heart sections for regeneration, with ‘class 1’ indicating complete regeneration showing contiguous ventricle wall, ‘class 2’ indicating partial regeneration showing some collagen deposition and ‘class 3’ indicating a block in regeneration showing fibrin and collagen deposition. Multiple sections of each heart were scored according to these criteria.

To quantify CM proliferation index, four to six sections showing the largest injury area were selected from each heart, and images were taken using a 10× objective. The number of Mef2^+^ and Mef2^+^ PCNA^+^ cells were manually counted using ImageJ software within a defined region including almost all Mef2^+^ PCNA^+^ cells around the injury site (1344×384 pixel region of the 1388×1040 pixel images). The percentages of Mef2^+^ PCNA^+^ cells from all sections were averaged to determine a proliferation index for each heart.

### qPCR analysis

For cDNA synthesis using PrimeScript II 1st Strand cDNA Synthesis Kit (TaKaRa), 1 µg of total RNA extracted from ventricles was used. RT-qPCR was carried out on biological triplicates with SYBR Premix Ex Taq II (Takara) using a Roche LightCycler 480 II system. The relative expression levels of target genes were normalized to *β-actin* and quantified by the 2^−ΔΔCT^ method (Livak and Schmittgen, 2001).

### RNA-seq and data analysis

Ventricles at 7 dpa were collected from 4-HT-treated *Tg(cmlc2:creER;cmlc2:nRSGG)* and *Tg(cmlc2:nRSGG)* animals, as well as *grl^5nt^*^−/−^ and WT sibling zebrafish. Six ventricles were pooled per biological replicate. Each group contained three biological replicates. Total RNAs of the ventricles were isolated using TRIzol Reagents (Life Technologies) and checked for a RIN number to inspect RNA integrity using the Agilent 2100 Bioanalyzer system (Agilent Technologies). Qualified total RNAs were further purified by RNAClean XP Kit (Beckman Coulter) and RNase-Free DNase Set (Qiagen). Libraries were constructed using the TruSeq RNA Sample Preparation Kit (Illumina) followed by a quality check with Qubit 2.0 Fluorometer (Invitrogen) and the Agilent 2100 Bioanalyzer system (Agilent Technologies), Sequencing was carried out on the Illumina HiSeq X Ten (Illumina) at Shanghai Biotechnology Corporation.

Sequenced raw reads were preprocessed using Seqtk to filter out rRNA reads, sequencing adapters, short-fragment reads and other low-quality reads to obtain clean reads for data analyses. Hisat2 (version 2.0.4) was used to map the clean reads to the zebrafish GRCz10 reference genome. After genome mapping, the mapped reads of genes were converted to FPKM (fragments per kilobase of exon model per million mapped reads) using Stringtie (version 1.3.0) (Pertea et al., 2016, 2015) for standardization of gene expression. The fold-changes of genes were calculated according to the FPKM in each sample. Genes with differential expression levels between samples were analyzed using edgeR (Robinson et al., 2010), with GO enrichment and KEGG pathway enrichment analyses.

### Transfection, ChIP and Luciferase assay

The Flag tag sequence fused with zebrafish *grl* CDS was ligated into the pEGFP-N1 vector. The plasmids were transfected into HEK 293T cells using Lipo 3000 Transfection Reagent (Invitrogen) as previously described (Ni et al., 2011). At 48 h after transfection, ChIP was performed using EZ-ChIP Kit (Millipore). HEK 293T cells were crosslinked with 1% formaldehyde for 10 min. After incubation with glycine to stop crosslinking, cells were rinsed in PBS and homogenized in SDS lysis buffer. Cell lysates were sonicated with a FS-300 Ultrasonic Processing apparatus. ChIP was then performed by using an antibody against Flag (mouse; Sigma) or a normal mouse IgG antibody (Millipore) as negative control, according to the manufacturer's instructions.

HEK 293T cells were cultured overnight at 8×10^4^ cells per well in 24-well plates. Firefly luciferase pGL3-*smyd2*-Fluc plasmid vector (0.5 µg) and *Renilla* luciferase pRL-TK plasmid vector (5 ng, as a normalization control) were co-transfected with different Grl mutant plasmids (0.5 µg) using 1 µl of Lipofectamine 2000 (Life Technologies) according to the manufacturer's protocol. Approximately 24 h later, cell lysates were collected and the firefly luciferase and *Renilla* luciferase activities measured using a Dual-Luciferase Reporter Assay System (Promega). The experiment was repeated three times.

### Western blot analysis for injured heart tissue

Ventricles from 4-HT-treated *Tg(cmlc2:creER;cmlc2:nRSGG)* and *Tg(cmlc2:nRSGG)* animals, as well as *grl^5nt^*^−/−^ and WT sibling zebrafish at 7 dpa were collected and homogenized with an electric homogenizer in cold RIPA lysis buffer (50 mM Tris pH 7.4, 150 mM NaCl, 1% Triton X-100 and 0.1% SDS) supplemented with proteinase inhibitors (Millipore) and phosphatase inhibitor cocktail (Apex Bio). The lysates were loaded into 12.5% SDS-PAGE after boiling for 5 min in SDS-PAGE sample loading buffer. Proteins were then transferred to PVDF membranes using a Trans-Blot Turbo Transfer System (Bio-Rad). Signals were detected by the Clarity Western ECL Substrate (Bio-Rad) and scanned using a ChemiScope series system (Clinx). Antibodies used for western blot in this study were as follows: anti-Smyd2 (Cell Signaling; #9734; 1:500), anti-P-Stat3 (Santa Cruz; sc-8059; 1:500), anti-Stat3 (Santa Cruz; sc-8019; 1:500), anti-methyl-lysine (Abbkine; ABM0060; 1:1000), anti-GAPDH (Abcam; ab181602; 1:10,000), anti-GAPDH (Abmart; M20006L; 1:4000), anti-actin (Sigma; A5441; 1:4000), anti-Flag (Sigma; 7425/1804; 1:4000), anti-Myc (HuaAn Biotechnology; R1208-1; 1:4000), anti-HA (Santa Cruz; sc-805; 1:4000), HRP-anti-rabbit IgG (Cwbio; CW0103S; 1:5000) and HRP-anti-mouse IgG (Cwbio; CW0102S; 1:5000).

### Co-IP assay

HEK 293T cells were transfected with pcDNA3-Flag-Stat3, pcDNA3-Myc-Smyd2a or pcDNA3-HA-Smyd2b plasmids and lysed 48 h later with IP lysis buffer (50 mM Tris-HCl pH7.5, 150 mM NaCl, 1% Triton X-100, 1 mM EDTA, 1× protease inhibitor cocktail and 1 mM DTT). The lysates were cleared by centrifugation at 13,600 ***g*** for 20 min at 4°C. The supernatants were diluted with binding buffer (50 mM Tris-HCl pH 7.5, 150 mM NaCl, 8% glycerol, 1 mM EDTA, 1× protease inhibitor cocktail and 1 mM DTT) to obtain a final Triton X-100 concentration of 0.2%, and then directly incubated with anti-Myc/HA-affinity beads for 4 h at 4°C. After centrifugation and washing three times with washing buffer (50 mM Tris-HCl pH 7.5, 150 mM NaCl, 0.1% Triton X-100, 1 mM EDTA, 1× protease inhibitor cocktail and 1 mM DTT), the protein-bead complexes were boiled in 1× SDS loading buffer and analyzed by western blot.

### Methylation and phosphorylation assay

For detection of methylation and phosphorylation of Stat3, HEK 293T cells were transfected with pcDNA3-Myc-Smyd2a, pcDNA3-HA-Smyd2b and pcDNA3-Flag-Stat3 as indicated. When inhibitors were used, cells were treated with AZ505 at different concentrations for 6 h before sample collection, whereas LLY-507 treatment was for 28 h. At 48 h post transfection, cells were washed with PBS twice and lysed in denaturing IP lysis buffer (150 mM NaCl, 0.1% NP40 or Triton X-100, 25 mM Tris-HCl, pH 8.0, 5 mM EDTA, 10% glycerol, 1% SDS, 1× protease inhibitor cocktail, 1× phosphatase inhibitor cocktail) and treated at 100°C for 20 min. The lysates were cleared by centrifugation at 13,600 ***g*** for 20 min. The supernatant was diluted with denaturing binding buffer without SDS to give a final SDS concentration of 0.1%, and then directly incubated with anti-Flag-M2-affinity beads (Bimake, Houston, TX) for 4 h. After extensive washing with IP washing buffer (50 mM Tris-HCl, pH 7.5, 150 mM NaCl, 0.1% Triton X-100, 1 mM EDTA), the immunoprecipitated proteins were boiled in 1× SDS loading buffer and analyzed by western blot.

### Imaging

AFOG and ISH images were taken using a Nikon Eclipse Ni microscope with a Nikon Digital Sight DS-Ri1 camera. Immunostaining images were taken using a Zeiss Axio Observer.Z1 microscope, Zeiss LSM 710 confocal microscope and Andor Dragonfly 500 High Speed confocal microscope.

### Statistical analysis

GraphPad software was used to perform statistical analysis. Data were analyzed by the two-tailed Student's *t*-test and Chi-square tests. For the Student's *t*-test, data are represented as mean±s.e.m. and considered significant at *P*<0.05.
